# David Versus Goliath: The Clinical Consequences of an Influenza A Viral Infection in an Unvaccinated Patient

**DOI:** 10.7759/cureus.4788

**Published:** 2019-05-31

**Authors:** Taylor S Harmon, Juan P Olano, Quan D Nguyen, Cihan Duran, Shaunak Patel

**Affiliations:** 1 Radiology, University of Florida College of Medicine, Jacksonville, USA; 2 Pathology, University of Texas Medical Branch, Galveston, USA; 3 Radiology, University of Texas Medical Branch, Galveston, USA

**Keywords:** diffuse alveolar damage, influenza a virus, influenza b virus, bacterial pneumonia, acute respiratory distress, polymerase chain reaction, extracorporeal membrane oxygenation, epidemic, vaccination, prevention

## Abstract

Acute influenza virus (AIV) infection can manifest as a severe life-threating illness in patients who are not vaccinated, and furthermore, have comorbidities that place them at risk for rapid respiratory decompensation. Each year influenza causes death in individuals with high risk for contracting this infection, although the illness is preventable by vaccination. Complications of AIV infection, such as bacterial pneumonia are treatable, but other severe complications such as acute respiratory distress syndrome (ARDS) leading to diffuse alveolar damage (DAD) are limited to supportive therapy and self-resolution. In most cases, ARDS leading to DAD is fatal, due to the insidious severity of symptoms which lead to rapid oxygen desaturation without correction, and despite supportive therapy. Regardless of a poor prognosis, the clinical signs and symptoms are congruent with imaging and attest to the importance of vaccination, which protect against high mortality rates.

## Introduction

Every year, influenza is observed by surveillance entities that exist within the local, state, and national levels. At each designated level, influenza virus is tracked and reported to better understand the disease and its progression throughout the winter season. In the winter of 2017 to 2018, the pathology department at the University of Texas Medical Branch (UTMB) in Galveston, Texas, observed the confirmed fatal cases of influenza. There were five cases of fatal acute influenza virus (AIV) infection in Galveston County, Texas, during that winter season that resulted in subsequent autopsies performed. Out of the five fatal cases of AIV reported at UTMB during that time, the cause of death for four of the cases were due to bacterial pneumonia superimposed to AIV, a common cause of mortality for patients with AIV infection. 

Three of the fatal AIV cases (60%) were the result of influenza A virus, and two of those cases (40%) were the result of influenza B virus. All AIV infections were confirmed by polymerase chain reaction (PCR). In two of the cases (33%), diffuse alveolar damage (DAD) was identified in the setting of acute respiratory distress syndrome (ARDS). DAD as a result of ARDS is often detrimental due to inadequate maintenance of oxygen saturation, and is often irreversible [1-2]. Though not the most common cause of mortality in patients with AIV infection, DAD and resultant ARDS is a much more serious etiology. The following case presents a patient with many comorbidities, that was found to have rapidly progressive DAD leading to ARDS, and ultimately resulting in their demise. This patient's case may be a testimony to the importance of vaccination, and how a rapidly progressive and high morality disease is completely preventable.

## Case presentation

**Figure 1:**
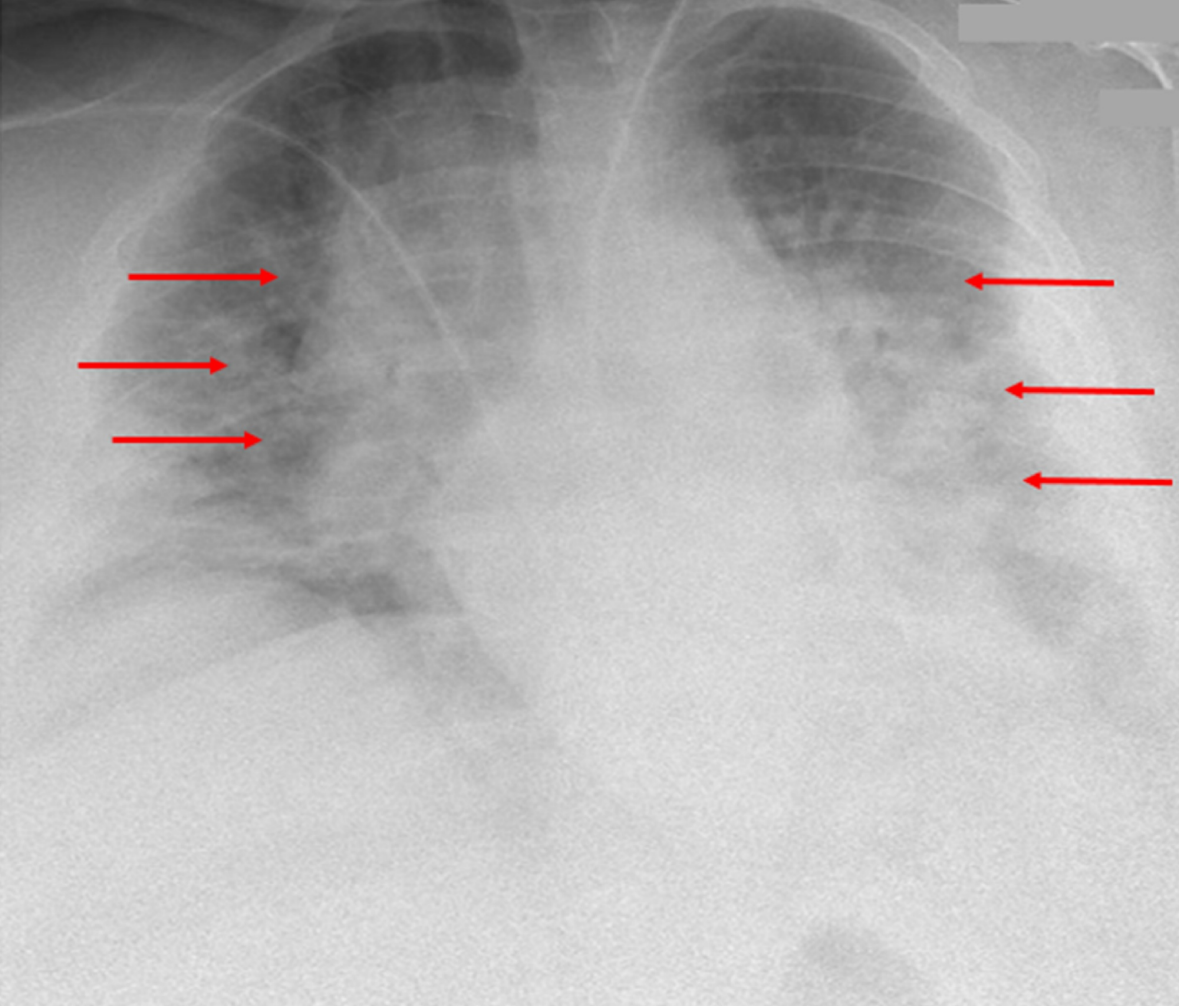
Initial chest radiograph An initial chest radiograph of the patient demonstrates diffuse and bilateral pulmonary opacities (red arrows), significant for acute respiratory distress syndrome. At this time, the etiology for acute influenza A virus is unknown, but the nidus of disease is suspected to be an infection.

A 41-year-old obese female with history of diabetes mellitus, morbid obesity, hypertension, seizure, asthma, obstructive sleep apnea, and anemia was admitted for acute respiratory failure to the intensive care unit. The patient had an initial oxygen saturation of 65, diarrhea, nausea, cough, and malaise for five days. The patient tested positive for influenza A via PCR, and a radiograph revealed underinflated pulmonary fields with bilateral airspace opacities, suggestive of ARDS with right hemidiaphragm elevation (Figure 1).

**Figure 2:**
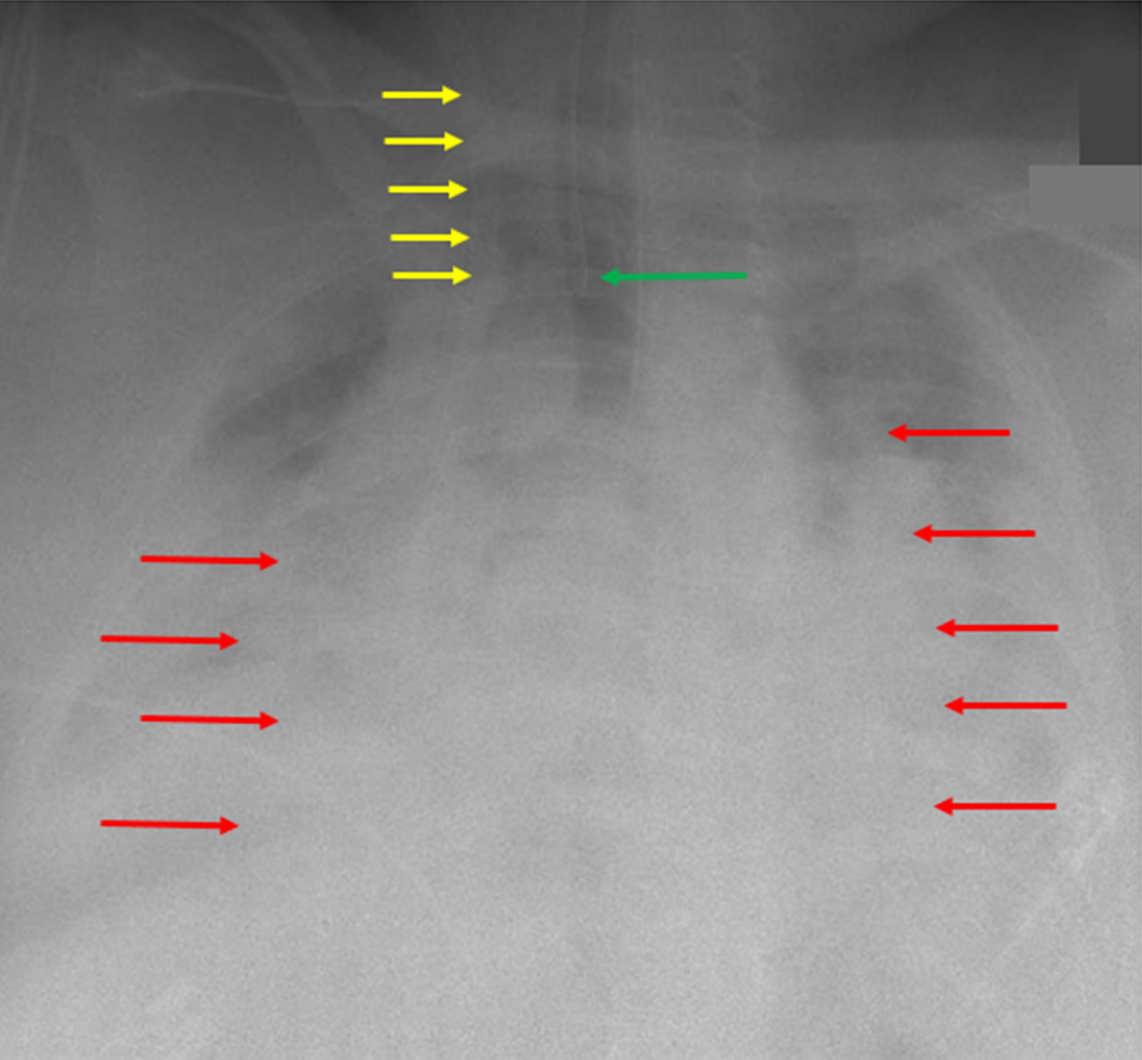
Follow-up radiograph after adjustment of the extracorporeal membrane oxygenation catheter A chest radiograph is shown after adjustment of the extracorporeal membrane oxygenation catheter (yellow arrows) in the right atrium of the heart. There is worsening bilateral pulmonary opacities (red arrows) present as a result of acute respiratory distress syndrome. The endotracheal tube (green arrow) can be seen appropriately placed just above the carina.

The patient was taken off bi-level positive airway pressure, and an endotracheal tube was placed just above the carina. Overnight, the patient’s oxygen saturation declined. The cardiothoracic surgery team was consulted, and extracorporeal membrane oxygenation (ECMO) was started for the patient. A 20 French catheter placement was confirmed on follow-up radiography (Figure 2).

**Figure 3:**
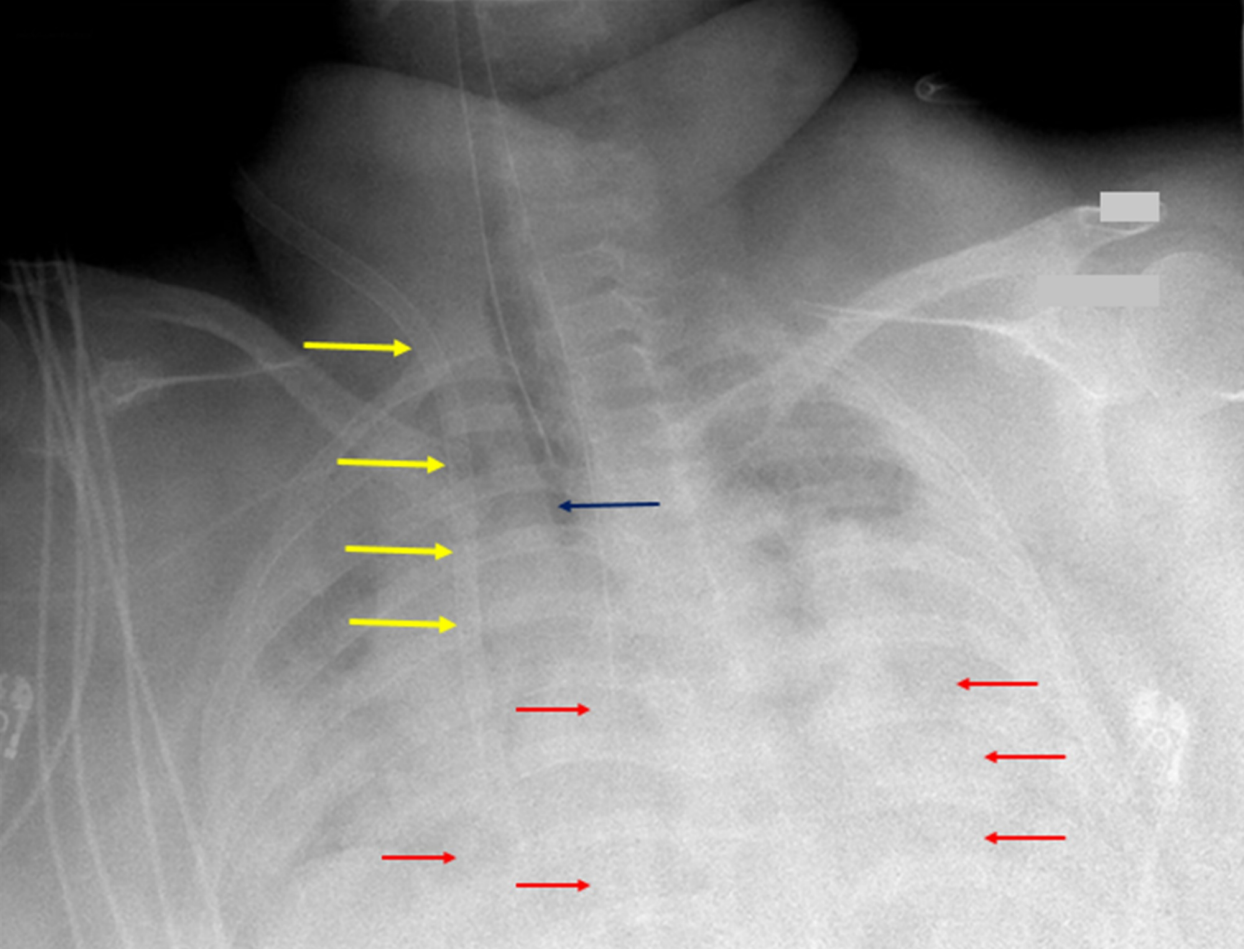
Final chest radiograph A final chest radiograph shows that pneumothoraces are not present. The imaging study continues to reveal persistent bilateral opacities (red arrows) as before, consistent with acute respiratory distress syndrome. A 20 French extracorporeal membrane oxygenation catheter is shown (yellow arrows) over the right lung field, terminating in the right atrium. The patient’s airway is patent, and is demonstrated by the dark blue arrow.

After two hours of ECMO therapy, the patient’s oxygenation did not improve. Subsequent radiographs as a result of ECMO catheter adjustment and confirmation for the absence of pneumothoraces demonstrated ARDS and worsening pulmonary edema. Upsizing of the ECMO catheter was attempted but resulted in cardiogenic shock once the circuit was clamped. The patient did not recover after attempted cardioversion, and a final chest radiograph revealed the persistence of diffuse ARDS (Figure 3).

**Figure 4:**
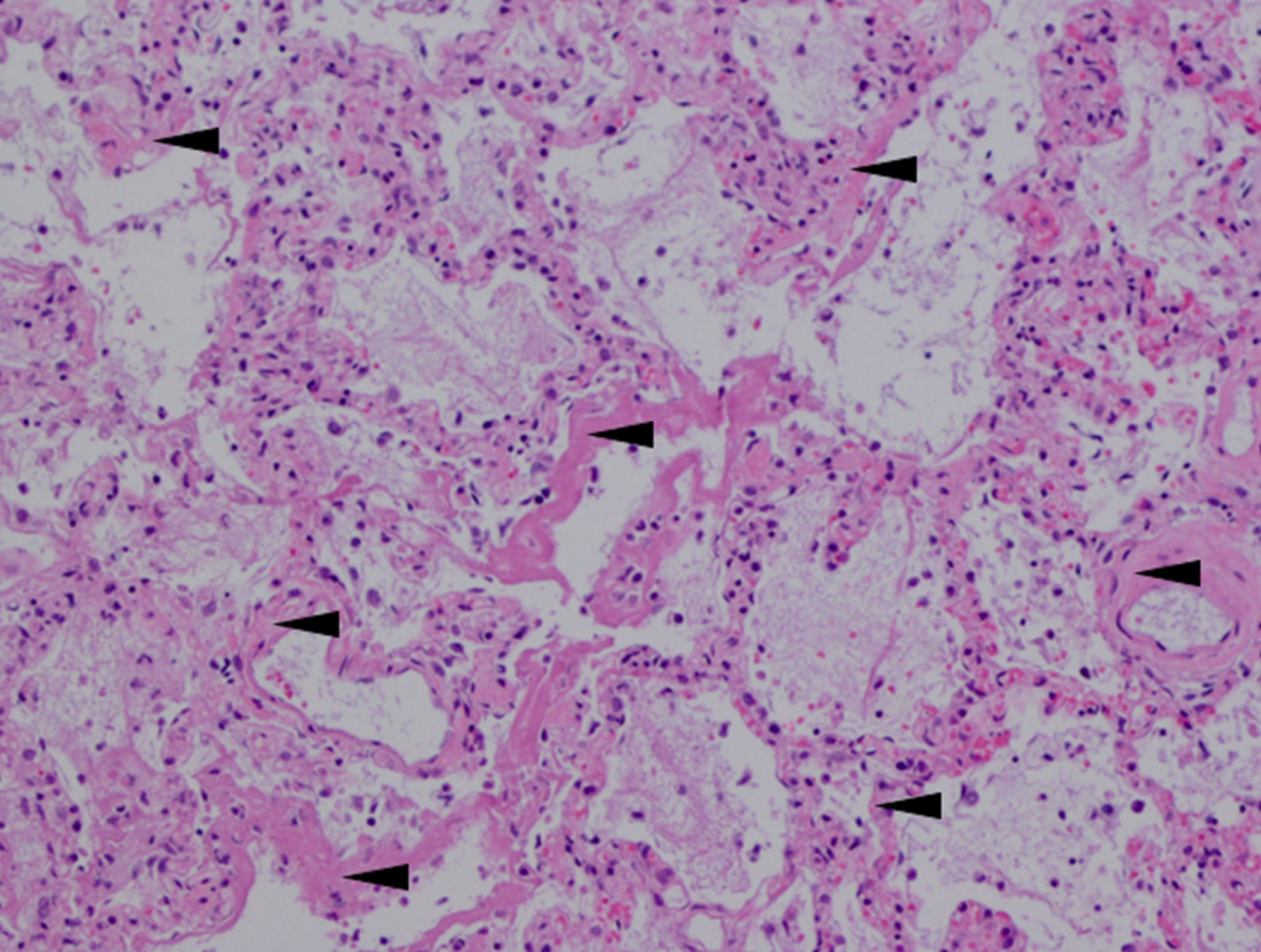
Autopsy results demonstrated by lung biopsy and hematoxylin and eosin staining A biopsy and hematoxylin and eosin stain of the patient’s lung tissue shows diffuse alveolar damage with characteristic hyalinization of the membrane and some exudative processes (black arrowheads). Hyalinization of the alveolar membranes is significant for acute respiratory distress syndrome. There is no evidence of superimposed bacterial pneumonia.

The autopsy results of the patient confirmed the presence of ARDS by demonstrating DAD without significant evidence for the superimposition of bacterial pneumonia (Figure 4).

Only 48 hours had elapsed from when the patient first presented to the emergency department at the start of her symptoms to the respiratory failure leading to her imminent demise. 

## Discussion

It is significant that the presence of AIV and rapidly progressive symptoms resulted in the demise of the patient, rather than from a more common etiology of mortality. Due to the overwhelming inflammatory response of the AIV infection and poor ventilation, the patient’s comorbid medical history such as diabetes mellitus, morbid obesity, hypertension, seizure, asthma, and obstructive sleep apnea, or all, could have been the result of the patient’s death [3-5]. Furthermore, of the five reported cases of AIV in Galveston county, Texas, during the 2017 to 2018 winter season, the patient reported was the youngest. This can suggest that the mortality threshold was decreased in this patient with many comorbidities, especially where influenza mortality remains mostly prevalent in children and the elderly [4].

Both patients with AIV A infection in Galveston county, Texas, during the 2017-2018 winter season demonstrated DAD, while cases due to AIV B infection only showed acute bacterial bronchopneumonia. The patient discussed had the most detrimental form of AIV infection to the extent of mortality, possibly before a superimposed bacterial bronchopneumonia could have formed. Most importantly, it is necessary to realize that regardless of the amount of attempts to resuscitate the patient, the annual influenza vaccine could have prevented the patient's demise.

Influenza related deaths reached record epidemic levels in the winter of 2017 to 2018 [6]. The synergistic combination of the preceding patient's comorbidities and their AIV A infection, resulting in their demise, reflects the severity of influenza related deaths during that winter season [7]. Vasopressors and bronchodilators had limited effect in opening the patient's airway, and oxygenation remained poor due to the massive accumulation of pulmonary edema and inflammatory response of ARDS [1-2]. In some patients with severe respiratory compromise, such as in this patient, ECMO is often utilized for oxygenation; however, ECMO therapy was limited due to the patient’s body habitus.

Precautions for influenza virus should be taken for children and the elderly, but do not negate the severity of disease in all other demographic groups [8]. Though rapidly progressive, DAD leading to ARDS has a high risk for mortality, in the case of this patient with multiple comorbidities for AIV infection, the influenza vaccination could have possibly saved her life. If possible, it should be applicable for everyone to receive the influenza vaccination every winter season.

## Conclusions

The presence of DAD due to ARDS in the case of AIV A infection is rapidly fatal; in the preceding case, the time between the onset of symptoms and the patient’s demise was only 48 hours. The patient's DAD in the setting of ARDS via AIV A infection poses a severe health risk, especially in patients with multiple comorbidities. Annual influenza vaccinations and reducing comorbid risk factors are preventative, especially in children and the elderly, individuals with high-risk profiles for the AIV infection, or both.
